# Remote cerebellar hemorrhage after the evacuation of a subdural hematoma: a case report

**DOI:** 10.11604/pamj.2022.41.24.32799

**Published:** 2022-01-11

**Authors:** Mohamed Amine Hadj Taieb, Kais Maamri, Amine Trifa, Ghassen Elkahla, Mohamed Maher Hadhri, Mehdi Darmoul

**Affiliations:** 1 Department of Neurosurgery, Fatouma Bourguiba Hospital, Monastir, Tunisia

**Keywords:** Postoperative, bleeding, cerebellar hemorrhage, zebra sign, case report

## Abstract

Remote intracranial hemorrhage is postoperative bleeding that occurs away from the surgical site. Remote cerebellar hemorrhage (RCH) is a cerebellar hemorrhage that may occur in 0.04-0.8% of cases after supratentorial and spinal procedures. We report a case of a 73-year-old male who developed signs of increased intracranial pressure two days after the evacuation of a subdural hematoma. Brain computed tomography showed RCH with the “zebra sign” and triventricular hydrocephalus that indicated the placement of external ventricle drain in emergency. Therefore, surgeons must pay special attention to this rare postoperative complication because it can be devastating in terms of patient outcome especially due to its possible complications requiring surgical treatment.

## Introduction

One of the major complications of craniotomy is a postoperative hemorrhage that usually occurs at the site of the operation. Remote cerebellar hemorrhage (RCH) is a rare postoperative complication after supratentorial procedures. It is characterized by spontaneous cerebellar bleeding after surgery for supratentorial or spinal pathology. This pathology is very rare and represents only 0.04% to 0.8% of postoperative complications in neurosurgery [1]. We present one of few cases of RCH after the evacuation for an acute subdural hematoma.

## Patient and observation

**Patient information:** a 73-year-old man with a medical history of diabetes and coronary disease was admitted to our emergency 2 hours after having a minor head trauma. He suffered from headaches and repetitive vomiting.

**Figure 1:**
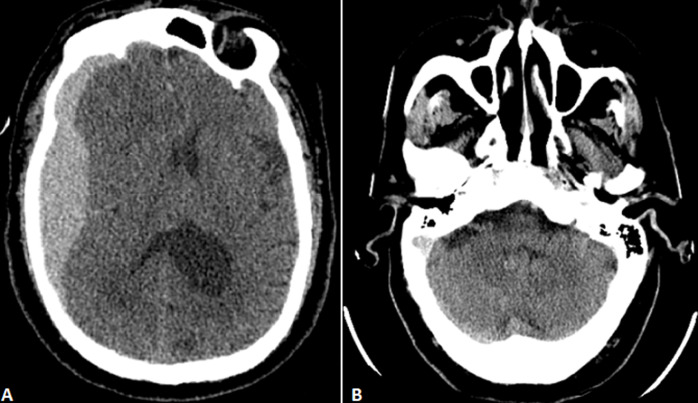
A) preoperative axial non-contrast CT scan showing a right-hemispheric acute subdural hematoma with right ventricular compression and midline-shift to the left; B) preoperative cerebellum view showing no abnormalities

**Clinical findings:** neurologic examination showed no motor weakness and no cerebellar signs. Computed tomography (CT) showed an acute subdural hematoma compressing the right cerebral hemisphere with no abnormality in the posterior fossa (Figure 1). He was not on antiplatelet or anticoagulant therapy. Preoperative coagulation parameters (prothrombin time (PT), international normalized ratio (INR), plated count) were normal. The patient underwent emergent surgery. He was treated with right frontoparietal craniotomy and evacuation.

**Timeline of the current episode:** the postoperative evolution was uneventful. Two days later, he presented acute headaches and vomiting.

**Figure 2:**
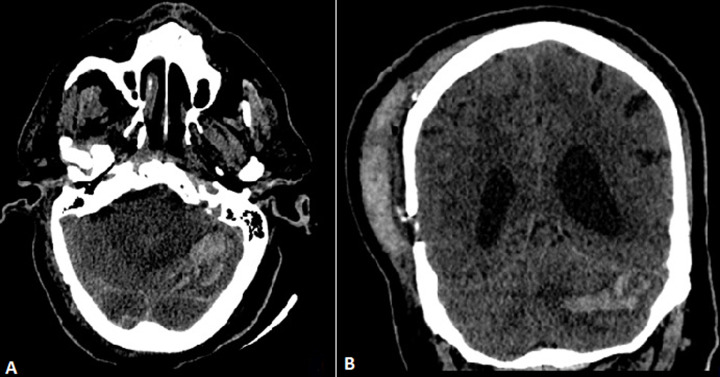
axial and coronal non-contrast CT scan of the brain on the second postoperative day revealed a cerebellar hemorrhage, dominating in the right cerebellar hemisphere and the vermis

**Diagnostic assessment and diagnosis:** computed tomography revealed a remote cerebellar hemorrhage with “a zebra sign” complicated with triventricular hydrocephalus (Figure 2).

**Therapeutic interventions:** we decided then to place an external ventricular drain (EVD) and the patient was admitted one day in intensive care.

**Follow-up and outcome of interventions:** one week later, after failed weaning attempt of EVD, we decided to place a ventriculoperitoneal shunt. The procedure went without any obvious complications. The patient was discharged from the hospital three days later. Follow-up examination, one year after the craniotomy, showed no neurological abnormalities.

**Informed consent:** the patient was informed of this manuscript and gave us his consent.

## Discussion

Although most postoperative intracranial hematomas generally occur at the site of the surgery, RCH is postoperative bleeding that occurs away from the surgical site, in the infratentorial compartment. It is a possible complication after supratentorial procedures (craniotomies, burr holes, or trans-sphenoidal procedures) [2] and even spinal surgery [3]. Its incidence in adult patients after supratentorial surgeries is 0.04-0.8% [1] with an exception for RCH after repair of unruptured anterior circulation aneurysms where the incidence is 3.5% [4,5]. In 2019, Tianqi Xu *et al*. investigated the rate of postoperative hemorrhage among 4588 craniotomies and they found nine cases of remote intracranial hemorrhage including only one case of RCH [6]. These numbers might be higher due to asymptomatic patients who do not undergo routine postop-erative imaging.

Until today, the exact pathophysiology of RCH remains widely debated and not fully elucidated. However, there is a consensus that the sudden decrease of intracranial pressure is the essential causative mechanism of remote intracranial hemorrhages [6]. Furthermore, some authors suggest that RCH seems to have a venous origin [7]. Several risk factors of RCH are divided into patient-dependent (hypertension, coagulopathy), intraoperative (head positioning, surgical approach, excessive cerebrospinal fluid (CSF) loss, induced coagulopathy), and postoperative factors (use of subdural/epidural drain, suction volume, patient positioning) [5]. In our case, there was no loss of CSF during the operation. Blood pressure, platelets count, and coagulation profiles were normal.

Most of the cases of RCH occurred immediately or within hours after the surgery. Clinically, RCH may present with signs of increased intracranial pressure and cerebellar signs. In 2006, Amini *et al*. [8] reported eight cases of RCH, three of which developed cerebellar symptoms, three were asymptomatic, one developed nausea, and one has passed away due to the poor prognosis following a head shotgun injury. Our patient had signs of increased intracranial pressure two days after the first surgery. Radiological features showed that RCH is located bilaterally (53.5%) as often as unilaterally (46.5%) [9]. In 2005, Brockmann *et al*. [10] have described an imaging appearance, named “Zebra sign”, on CT in patients with RCH. This sign refers to a pattern, comparable to zebra skin, of hyperdensity indicative of blood and hypodensity indicative of normal cerebellar parenchyma in a curvilinear, stripe-like fashion along the cerebellar folia. Zebra sign was found in 64% of cases in a systematic review of RCH after supratentorial procedures [2].

Treatment of RCH is dependent on the clinical picture and imaging findings. Most cases resolve spontaneously. Brockmann *et al*. [10] conducted a meta-analysis of RCH revealed a good overall prognosis with no remaining deficits in 32.3 [9]. However, many devastating complications may arise after RCH which may require urgent operation such as acute hydrocephalus. This is similar to our case, where an external ventricle drain was placed. Not one single preoperative or surgical factor can reliably predict the occurrence of cerebellar hemorrhage after supratentorial craniotomy. The only preventive measure is to avoid excessive loss of CSF.

## Conclusion

Remote cerebellar hemorrhage is a rare postoperative complication. In the majority of cases, it is benign but can be devastating especially because of its complications requiring emergency life-saving surgery. Many risk factors seem to be associated with RCH. The only adjustable factor related to RCH is the loss of CSF during and after surgery.
